# Service Perceptions in Fitness Centers: IPA Approach by Gender and Age

**DOI:** 10.3390/ijerph17082844

**Published:** 2020-04-21

**Authors:** Jairo León-Quismondo, Jorge García-Unanue, Pablo Burillo

**Affiliations:** 1 Faculty of Sport Sciences, Universidad Europea de Madrid, 28670 Madrid, Spain; pablo.burillo@universidadeuropea.es; 2 IGOID Research Group, Department of Physical Activity and Sport Sciences, University of Castilla-La Mancha, 45071 Toledo, Spain; jorge.garciaunanue@uclm.es

**Keywords:** fitness centers, importance-performance analysis (IPA), correlation, gender, age

## Abstract

Background: The number of fitness practitioners has increased in the last decades. A deeper understanding of user perceptions is required for better service design. Methods: An importance-performance analysis (IPA) and correlational analysis were performed on a sample of 414 members (173 women and 241 men) with a mean age of 32.33 years (SD = 11.50) and recruited from 25 fitness centers of Community of Madrid, Spain. Results: The results show that women’s levels of importance and performance are higher than men in most of the service attributes. Women also correlate with a higher priority than men in core elements of the service, such as the variety and number of activities, personal training and fitness service quality. Female members feel more attracted by services like swimming pools and other peripheral services, like a welcome pack and medical or physiotherapist service. According to age, older members feel less satisfied than young users with the cleanliness of activity spaces and with the safety of lockers. Conclusions: Differences in perceptions by age and gender were identified among members of fitness centers. These results should be considered by private and public organizations to provide the best practices and tailored services for engaging more people in physical activity.

## 1. Introduction

In recent years, global increasing interest in physical activity and sports practice has happened. Consequently, the number of services related to well-being, physical activity or sports has grown, aiming to respond to such demand [1]. Thus, physical activity participants have changed from a situation in which they had to adapt to the offer of sports services to the opposite one. Nowadays, physical activity offer is much diverse and is more tailored to the user’s needs [2]. In this regard, fitness centers are a reference of health services, helping to encourage people to engage in physical activity services [3,4,5]. The open and dynamic nature of the fitness industry [6] means that it is continually evolving, adapting to every situation and context. Therefore, users have more possibilities to compare services in order to choose the one that best meets their needs. Hence, sports services providers should consider customers as expert [7].

The research field has attracted attention in the last decades, including those exploring service quality [8]. Perceived quality [9,10,11,12], satisfaction [13,14,15,16] and loyalty [17,18,19] are some examples of the research lines related to the fitness industry in last years. All this information is considered essential for a better understanding of consumers, for offering highly professional service and for better practices in fitness centers. However, an apparent gap in the literature regarding practical applications of tools in the fitness industry is identified.

**Figure 1:**
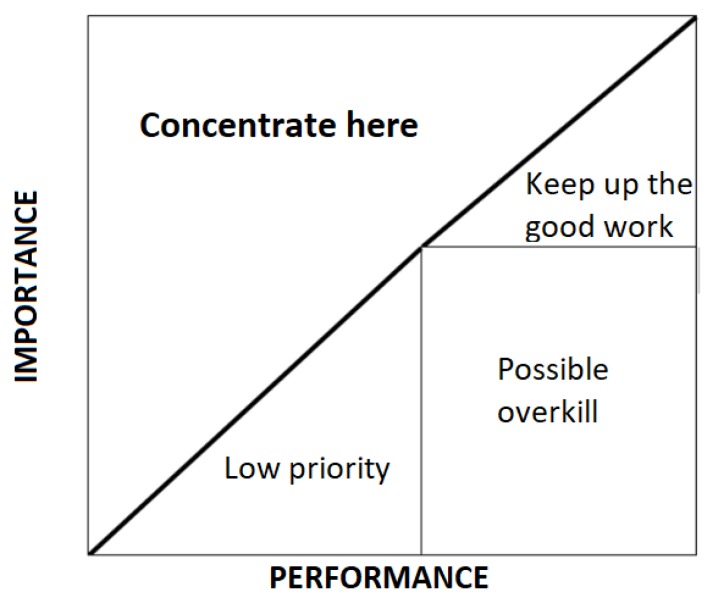
Importance-performance analysis (IPA) matrix.

The relationship between research and practices is progressively becoming closer. A detailed analysis of the provided service contributes to make managerial actions more precise, by taking into consideration some variables, such as demographic factors [20,21]. Thus, importance-performance analysis (IPA), developed by Martilla and James [22], is a useful tool for any kind of organization. It has recently been applied in health clubs, fitness, well-being services [9,12,23,24,25,26,27,28,29]. IPA is based on respondents’ judgments on the importance and performance of different service attributes. This way, it allows differentiating the strengths and the weakens. Following Ábalo, Varela, and Rial [30], according to the level of importance and performance, every attribute can be classified into concentrate here, keep up the good work, possible overkill or low priority (Figure 1). This makes possible to understand the consumer priorities, perceptions and, subsequently, the more urgent areas to attend to.

In IPA, the discrepancy between the level of performance and importance is also measured. A negative discrepancy is related to levels of dissatisfaction, whereas a positive one leads to satisfaction. In the IPA matrix, the discrepancy line is represented as a crossing line, also understood as iso-rating or iso-priority line [31]. Thus, larger positive discrepancies lead to greater satisfaction, while larger negative discrepancies cause greater dissatisfaction.

The purpose of this work is to discover the differences in perceptions and priorities regarding gender and age. Even though some studies on physical activity participation and sports performance have been completed, the knowledge of physical activity participation motives and perceptions—specifically in fitness centers—should be examined. In this way, public and private sports organizations could promote politics highly related to customer needs, according to their gender and age.

Thus, the aim of this paper is to explore the perceptions of fitness services quality by gender and age, as well as to examine the correlation of different service attributes with these two variables.

## 2. Materials and Methods

### 2.1. Design and Participants

**Figure 2:**
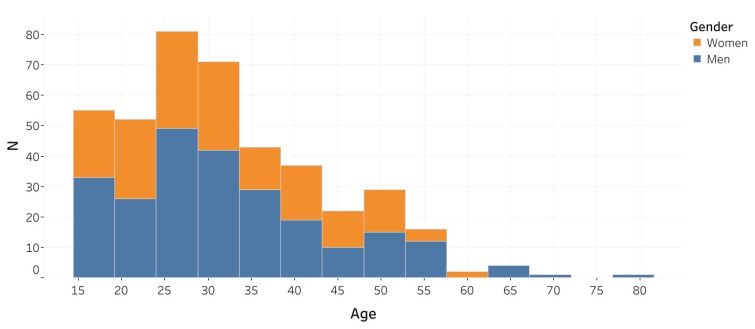
Histogram of age by gender.

A correlational study was conducted with a convenience sample of 414 members (Figure 2) recruited from 25 privately managed fitness centers, including 8 low-cost centers (*n* = 148 members), 15 mid-market centers (*n* = 212 members), and 2 premium centers (*n* = 54 members). Participants were aged between 18 and 77 (M = 32.33 years; SD = 11.50) (Table 1). Most of the participants had belonged to the center for longer than 12 consecutive months (56.30%), had a university certification (55.60%), and they were closer than 15 minutes to the fitness center (84.10%). Peak attendance time was on Mondays between 6 p.m. and 10 p.m.

All the participants should belong to privately managed fitness centers located in the Community of Madrid, Spain, not oriented to a simple sport modality or martial arts, with at least one weight room with machines and free weight, and one or more rooms for group classes. Although it was not a mandatory requirement, 11 fitness centers had a swimming pool.

### 2.2. Instrument

A validated questionnaire based on importance-performance analysis (IPA) [22] was designed. The questionnaire validation was performed in Spain. An initial literature review allowed to determine the most useful information to collect [13,15,24,32,33,34]. Next, the number of initial items was reduced and, later, reviewed by a panel of experts (four PhD university professors with a research experience of more than five years and two managers of fitness centers with more than five years of experience). The panel of experts offered their individual opinion about the adequacy of each item. Finally, an agreement was reached. The final version comprises 29 items for the importance scale (*α* = 0.873) and the same 29 elements for the performance scale (*α* = 0.895).

Every participant valued on a 5-point Likert scale the level of importance of the 29 attributes (1 = not important; 5 = very important), as well as the level of performance of the same 29 attributes (1 = low performance; 5 = high performance).

### 2.3. Procedure

Questionnaires were distributed personally by the same researcher, between June of 2016 and November 2017. This extensive period of data collection allowed a wide variety of participants to be covered, with diverse characteristics. The contact with participants was either at the moment of arriving or leaving the fitness center. All of them were informed about the research objectives and they accepted to participate voluntarily.

### 2.4. Data Analysis

The analysis was performed with IBM SPSS 23.0 Statistics software (IBM Inc., Chicago, IL, USA), setting the critical level of significance at *p* < 0.05. The descriptive data are described as mean and standard deviation. Kolmogorov Smirnoff analysis showed a non-normal behavior of variables. Therefore, non-parametric tests were performed. A Wilcoxon test was used for inference analysis in comparisons between importance and performance, while a Spearman test was applied for correlational analyses. The sociodemographic variables considered were gender and age. Regarding the IPA model, the data interpretation was based on Ábalo et al. [30].

## 3. Results

### 3.1. Gender

The IPA analysis contrast between men and women is displayed in Table 2. Overall results show that women’s levels of importance and performance are higher than the men’s. However, given the average level of discrepancy, the perception of female members is related to higher levels of dissatisfaction (women = −0.60; men = −0.52).

**Figure 3:**
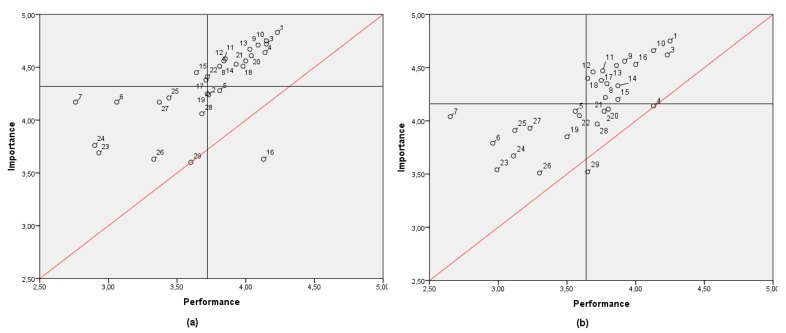
IPA matrix comparison by gender: (**a**) women; (**b**) men.

Figure 3 shows the IPA matrix by gender, presenting the aggregate importance and performance values. It can be observed that most of the analyzed items are in the *concentrate here* area, above the discrepancy line. This means all these elements are related to levels of dissatisfaction. A possible waste of resources is present in item 16 for women, spacious and hygienic restrooms, and item 29 for men, fitness center as a social meeting point.

Table 3 illustrates the correlation between gender and both, importance and performance values. Statistically significantly higher levels in both scales, importance and performance, positively correlate with female members.

### 3.2. Age

**Figure 4:**
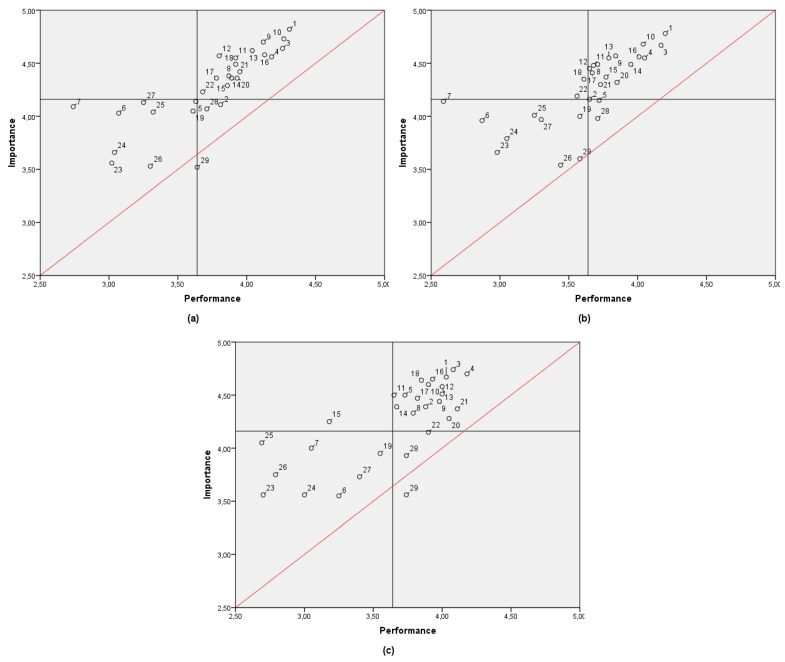
IPA matrix comparison by age: (**a**) 18 to 29; (**b**) 30 to 49; (**c**) 50 to 77.

The same analysis was performed by age. Three groups were considered: 18 to 29 years old (*n* = 202), 30 to 49 years old (*n* = 168), and 50 to 77 years old (*n* = 44). Figure 4 shows the IPA results for each group. The data distribution is more similar between the young and middle-age groups, but some contrast is represented in the older group.

Table 4 shows the correlation between age and both, importance and performance values. Statistically significant correlations can be observed. The age shows a negative correlation with many importance and performance items. A significantly negative correlation is observed in performance scale, items 10 and 18. No significantly positive correlation exists.

## 4. Discussion

The main results show, through importance-performance analysis (IPA), different perceptions and priorities for members of fitness centers.

According to gender, the level of importance and performance is usually higher among female members. In general, men and women target the same service attributes. However, in view of the IPA and the correlational analysis, a statistically significant correlation between females and higher importance and performance values is revealed.

Firstly, female members correlate with higher levels of importance of the variety and number of activities, as well as they correlate with a better perception of that attribute’s performance. The same is the case of the fitness service quality, which correlates with higher expectations among female members, but also with a better perception. These results show that the core service is essential, especially for women. These data are coherent with previous findings, regarding that preference for fitness group classes is higher among females, representing up to 74.50% of participants [35,36,37,38,39,40]. This is a significant fact, given that group training is in the third position in the worldwide fitness trends for 2020 [41].

Higher levels of importance of personal training service and kind treat from staff also correlate with the female gender. In this regard, the level of satisfaction with the achievement of outcomes thanks to instructors is better perceived by female members. Considering previous investigations on women’s experiences with personal training [42], a positive experience consisting of relationships, trainer qualities, outcomes and motivation is fundamental for female members.

The level of importance of the swimming pool in general and, specifically its temperature, is directly correlated with female members. This also happens with the importance of hair dryers in restrooms, something consistent with the usual needs of each group. It is remarkable that there is evidence that women are more likely to practice recreational swimming in search of psychological well-being, whereas men focus on performance. For that reason, the swimming pool and its temperature seem to show higher values among women.

Regarding marketing strategies, women correlate with receiving gifts when joining the center. In the case of men, this is not happening to the same extent. Lastly, the importance of medical or physiotherapist services is also statistically significantly correlated with female members. Although those two last elements are not a fundamental part of the service, they should be considered by managers of fitness centers, helping them to tailor the service provided to both women and men.

Regarding IPA and correlational analysis by age, two items are statistically significantly correlated. The performance of hygiene and cleanliness of activity spaces show a significantly positive correlation with age, meaning that older people are less satisfied with the hygiene and cleanliness of those spaces. In sports services, different processes and interactions between members and the physical environment have been studied [43,44,45]. Members of fitness centers are increasing their expertise in the industry [7]. Although generally, the intangible aspect of fitness centers services is fundamental, some researches remark the importance of the tangible aspects, given that members are familiar with the fitness industry and the physical context around them [44].

In addition, age is also negatively correlated with the performance of spacious and safe lockers. Other researches have studied the quality of the locker service and the locker system from the point of view of managers of fitness centers [29]. Those elements are not considered priority from the managers’ perspective. However, they should consider a safer environment especially when the target consumer is 50 years old or older.

## 5. Conclusions

This paper offers a useful tool for measuring the expectations and perceptions in fitness centers. Through importance-performance analysis (IPA), it was found that both women and men target the same priorities, but women give more importance to core elements of the service such as the variety and number of activities, personal training and fitness service quality. In addition, they feel more attracted by a fitness center with swimming pool and when they are provided peripheral services, like a welcome pack, medical or physiotherapist services. Furthermore, older members feel less satisfied with the hygiene and cleanliness of activity spaces are hygienic, as well as with the safety of lockers. Decisions regarding the quality of fitness centers services should consider this information, helping to offer a tailored experience and promoting the participation and engagement in physical activity services.

## Figures and Tables

**Table 1:** Mean age by gender.

Gender	*N*	Age in Years	SD
Women	173	31.92	11.00
Men	241	31.62	11.85

Annotations: *N* = frequency; SD = Standard deviation.

**Table 2:** IPA results according to gender.

	Items	Women					Men				
		I	SD	P	SD	D	I	SD	P	SD	D
1	Accessibility: closeness to member homes	4.83	0.53	4.23	1.08	−0.60 **	4.75	0.67	4.25	0.91	−0.50 **
2	Fees	4.24	1.02	3.73	1.06	−0.51 **	4.09	1.10	3.77	0.94	−0.32 **
3	Hours and days of operation	4.72	0.74	4.15	1.21	−0.57 **	4.62	0.76	4.23	0.97	−0.42 **
4	Kindness and treat from staff	4.64	0.64	4.14	1.03	−0.50 **	4.14	1.00	4.13	0.87	−0.01 **
5	Achievement of outcomes from instructors	4.28	0.88	3.81	1.04	−0.47 **	4.09	1.00	3.56	0.96	−0.53 **
6	Gifts for attracting new customers	4.17	1.03	3.06	1.12	−1.11 **	3.79	1.19	2.96	1.12	−0.83 **
7	Gifts for rewarding current customers	4.17	0.99	2.76	1.09	−1.41 **	4.04	0.99	2.65	1.07	−1.39 **
8	Large activity spaces	4.51	0.87	3.81	0.98	−0.70 **	4.35	0.94	3.79	0.98	−0.56 **
9	Level of maintenance of activity spaces	4.71	0.53	4.09	0.97	−0.62 **	4.56	0.81	3.92	1.00	−0.64 **
10	Hygiene and cleanliness of activity spaces	4.75	0.57	4.15	0.95	−0.60 **	4.66	0.77	4.13	0.93	−0.53 **
11	Ventilation (temperature) of activity spaces	4.56	0.80	3.84	1.07	−0.72 **	4.47	0.85	3.76	1.09	−0.71 **
12	Number of pieces of equipment	4.58	0.75	3.85	1.00	−0.73 **	4.46	0.94	3.69	1.00	−0.77 **
13	Level of maintenance of equipment/material	4.67	0.60	4.03	0.93	−0.64 **	4.52	0.79	3.86	1.02	−0.66 **
14	Swimming pool/SPA (general)	4.53	0.94	3.93	1.15	−0.60 **	4.33	1.06	3.87	1.06	−0.46 **
15	Swimming pool temperature	4.45	0.97	3.64	1.17	−0.81 **	4.20	1.05	3.87	1.07	−0.33 **
16	Spacious and hygienic restrooms	3.63	0.72	4.13	0.92	+0.50 **	4.53	0.75	4.00	0.98	−0.53 **
17	Shower quality	4.38	0.92	3.71	1.01	−0.67 **	4.38	0.91	3.75	1.04	−0.63 **
18	Spacious and safe lockers	4.51	0.83	3.98	0.98	−0.53 **	4.40	0.90	3.65	1.08	−0.75 **
19	Hair dryer in restrooms	4.25	1.03	3.72	1.15	−0.53 **	3.85	1.10	3.50	1.12	−0.35 **
20	Variety and number of activities	4.61	0.70	4.04	0.92	−0.57 **	4.11	0.95	3.80	0.94	−0.31 **
21	Fitness service quality	4.56	0.70	4.00	0.93	−0.56 **	4.22	0.94	3.78	0.90	−0.44 **
22	Personal training quality	4.41	0.97	3.72	1.09	−0.69 **	4.05	1.02	3.59	0.97	−0.46 **
23	Lending towels service	3.69	1.27	2.93	1.05	−0.76 **	3.54	1.18	2.99	1.08	−0.55 *
24	Personal hygiene products in restrooms	3.76	1.27	2.90	1.06	−0.86 **	3.67	1.15	3.11	1.17	−0.56 **
25	Medical/physiotherapist services	4.21	1.05	3.44	0.94	−0.77 **	3.91	1.13	3.12	1.15	−0.79 **
26	Cafeteria/restaurant	3.63	1.31	3.33	1.21	−0.30	3.51	1.16	3.30	1.07	−0.21 *
27	Wi-Fi	4.17	1.13	3.37	1.32	−0.80 **	3.93	1.29	3.23	1.40	−0.70 **
28	Client profile in the fitness center	4.06	0.94	3.68	0.90	−0.38 **	3.97	0.97	3.72	0.82	−0.25 **
29	Fitness center as social meeting point	3.60	1.20	3.60	0.93	0.00	3.52	1.25	3.65	0.91	+0.13
	Mean	4.32	−	3.72	−	−0.60	4.16	−	3.64	−	−0.52

Annotations: I = importance; P = performance; D = discrepancy; SD = standard deviation; * *p* < 0.05; ** *p* < 0.01.

**Table 3:** Gender correlation with importance and performance scores.

	Items	Importance	Performance
		Correlation with Gender	Correlation with Gender
1	Accessibility: closeness to member homes	0.056	0.036
2	Fees	0.069	−0.001
3	Hours and days of operation	0.104 *	0.022
4	Kindness and treat from staff	0.098 *	0.043
5	Achievement of outcomes from instructors	0.089	0.140 **
6	Gifts for attracting new customers	0.160 **	0.041
7	Gifts for rewarding current customers	0.029	0.048
8	Large activity spaces	0.097 *	0.015
9	Level of maintenance of activity spaces	0.067	0.090
10	Hygiene and cleanliness of activity spaces	0.028	0.020
11	Ventilation (temperature) of activity spaces	0.056	0.038
12	Number of pieces of equipment	0.086	0.082
13	Level of maintenance of equipment/material	0.087	0.090
14	Swimming pool/SPA (general)	0.109 *	0.054
15	Swimming pool temperature	0.145 **	−0.092
16	Spacious and hygienic restrooms	0.089	0.064
17	Shower quality	0.012	−0.023
18	Spacious and safe lockers	0.064	0.153 **
19	Hair dryer in restrooms	0.191 **	0.110 *
20	Variety and number of activities	0.292 **	0.148 **
21	Fitness service quality	0.186 **	0.134 **
22	Personal training quality	0.204 **	0.078
23	Lending towels service	0.070	−0.017
24	Personal hygiene products in restrooms	0.056	−0.090
25	Medical/physiotherapist services	0.147 **	0.137
26	Cafeteria/restaurant	0.058	0.020
27	Wi-Fi	0.098	0.044
28	Client profile in the fitness center	0.039	−0.013
29	Fitness center as social meeting point	0.023	−0.035

Annotations: * *p* < 0.05; ** *p* < 0.01.

**Table 4:** Age correlation with importance and performance scores.

	Items	Importance	Performance
		Correlation with Age	Correlation with Age
1	Accessibility: closeness to member homes	−0.049	−0.050
2	Fees	0.088	−0.021
3	Hours and days of operation	0.040	−0.014
4	Kindness and treat from staff	0.071	0.001
5	Achievement of outcomes from instructors	0.085	0.082
6	Gifts for attracting new customers	−0.085	−0.042
7	Gifts for rewarding current customers	0.010	−0.013
8	Large activity spaces	0.045	−0.029
9	Level of maintenance of activity spaces	−0.074	−0.075
10	Hygiene and cleanliness of activity spaces	−0.031	−0.122 *
11	Ventilation (temperature) of activity spaces	−0.032	−0.091
12	Number of pieces of equipment	−0.040	0.008
13	Level of maintenance of equipment/material	−0.063	−0.086
14	Swimming pool/SPA (general)	0.047	−0.041
15	Swimming pool temperature	0.018	−0.118
16	Spacious and hygienic restrooms	0.014	−0.062
17	Shower quality	0.046	−0.018
18	Spacious and safe lockers	−0.001	−0.102 *
19	Hair dryer in restrooms	−0.012	−0.008
20	Variety and number of activities	−0.014	−0.003
21	Fitness service quality	−0.051	−0.034
22	Personal training quality	−0.035	0.013
23	Lending towels service	0.023	−0.036
24	Personal hygiene products in restrooms	0.016	0.018
25	Medical/physiotherapist services	0.005	−0.101
26	Cafeteria/restaurant	0.034	−0.027
27	Wi-Fi	−0.095	0.023
28	Client profile in the fitness center	−0.043	0.012
29	Fitness center as social meeting point	0.023	−0.002

Annotations: * *p* < 0.05.
